# Fixation of intraoperative proximal femoral fractures during THA using two versus three cerclage wires - a biomechanical study

**DOI:** 10.1186/s12891-021-04956-5

**Published:** 2022-01-07

**Authors:** Toni Wendler, Melanie Edel, Robert Möbius, Johannes Fakler, Georg Osterhoff, Dirk Zajonz

**Affiliations:** 1 ZESBO - Center for Research on Musculoskeletal Systems, Leipzig University, Semmelweisstraße 14, 04103 Leipzig, Germany; 2 Institute of Anatomy, Leipzig University, Leipzig, Germany; 3 Department of Orthopaedic, Trauma and Plastic Surgery, Leipzig University, Leipzig, Germany; 4 Department of Neurosurgery, Leipzig University, Leipzig, Germany; 5 Department of Orthopaedic, Trauma and Plastic Surgery, Zeisigwaldkliniken Bethanien Chemnitz, Chemnitz, Germany

**Keywords:** Intraoperative fracture, Proximal femoral fracture, Cerclage wiring, Cementless stem, Total hip arthroplasty, Biomechanical study

## Abstract

**Background:**

Intraoperative proximal femoral fractures (IPFF) are relevant complications during total hip arthroplasty. Fixation using cerclage wires (CW) represents a minimally-invasive technique to address these fractures through the same surgical approach. The goal of treatment is to mobilise the patient as early as possible, which requires high primary stability. This study aimed to compare different cerclage wire configurations fixing IPFF with regard to biomechanical primary stability.

**Methods:**

Standardised IPFF (type II, Modified Mallory Classification) were created in human fresh frozen femora and were fixed either by two or three CW (1.6 mm, stainless steel). All cadaveric specimens (*n* = 42) were randomised to different groups (quasi-static, dynamic) or subgroups (2 CW, 3 CW) stratified by bone mineral density determined by Dual Energy X-ray Absorptiometry. Using a biomechanical testing setup, quasi-static and dynamic cyclic failure tests were carried out. Cyclic loading started from 200 N to 500 N at 1 Hz with increasing peak load by 250 N every 100 cycles until failure occurred or maximum load (5250 N) reached. The change of fracture gap size was optically captured.

**Results:**

No significant differences in failure load after quasi-static (*p* = 0.701) or dynamic cyclic loading (*p* = 0.132) were found between the experimental groups. In the quasi-static load testing, all constructs resisted 250% of the body weight (BW) of their corresponding body donor. In the dynamic cyclic load testing, all but one construct (treated by 3 CW) resisted 250% BW.

**Conclusions:**

Based on this in vitro data, both two and three CW provided sufficient primary stability according to the predefined minimum failure load (250% BW) to resist. The authors recommend the treatment using two CW because it reduces the risk of vascular injury and shortens procedure time.

**Supplementary Information:**

The online version contains supplementary material available at 10.1186/s12891-021-04956-5.

## Background

Total hip arthroplasty (THA) is considered one of the most successful surgical interventions [1]. However, even this frequently performed operation is associated with complications. Femoral fractures like fissures of the proximal femur are a frequent intraoperative complication [2], especially in the presence of limited bone quality (osteopenia or osteoporosis) in geriatric patients [3, 4]. There is a trend away from cemented towards uncemented femoral stem fixation, further increasing the risk of intraoperative proximal femoral fractures (IPFF) [5, 6]. The incidence rate of IPFF is reported to range from 1.0 to 3.2% [2, 4, 5, 7]. Even higher incidences of 2.2 to 13.4% are reported with the use of cementless Spotorno stems [8] as a result of their straight and tapered design as well as the associated proximal and metaphyseal load application to the bone. Conservative management is not appropriate in most cases, especially in case of uncemented implantation, due to the risk of crack propagation or fracture displacement [9] which often leads to prolonged bone healing process and immobilisation [10] as well as high costs incurred by the health care system [11]. For the mostly geriatric patient population, IPFF can have further serious consequences like an increased risk of nosocomial infections, thromboembolic events, and mortality due to aggravated or delayed mobilisation [12, 13]. Therefore, the goal of treatment is to achieve fracture fixation with high primary stability in order to allow patient’s early mobilisation. Preferably, this fixation can be done through the existing surgical approach that was chosen for implantation of THA components. For these reasons, cerclage wiring has become an established fixation option to stabilise IPFF [14, 15]. It can be assumed that a larger number of cerclage wires (CW) will provide higher primary stability. However, there is a risk that CW can pinch or injure blood vessels [16], why the number of CW used should be as small as possible in order to minimise this risk while attaining the same stability. Therefore, the purpose of this study is to evaluate the primary stability of fixed IPFF using different numbers of CW.

## Methods

**Fig. 1:**
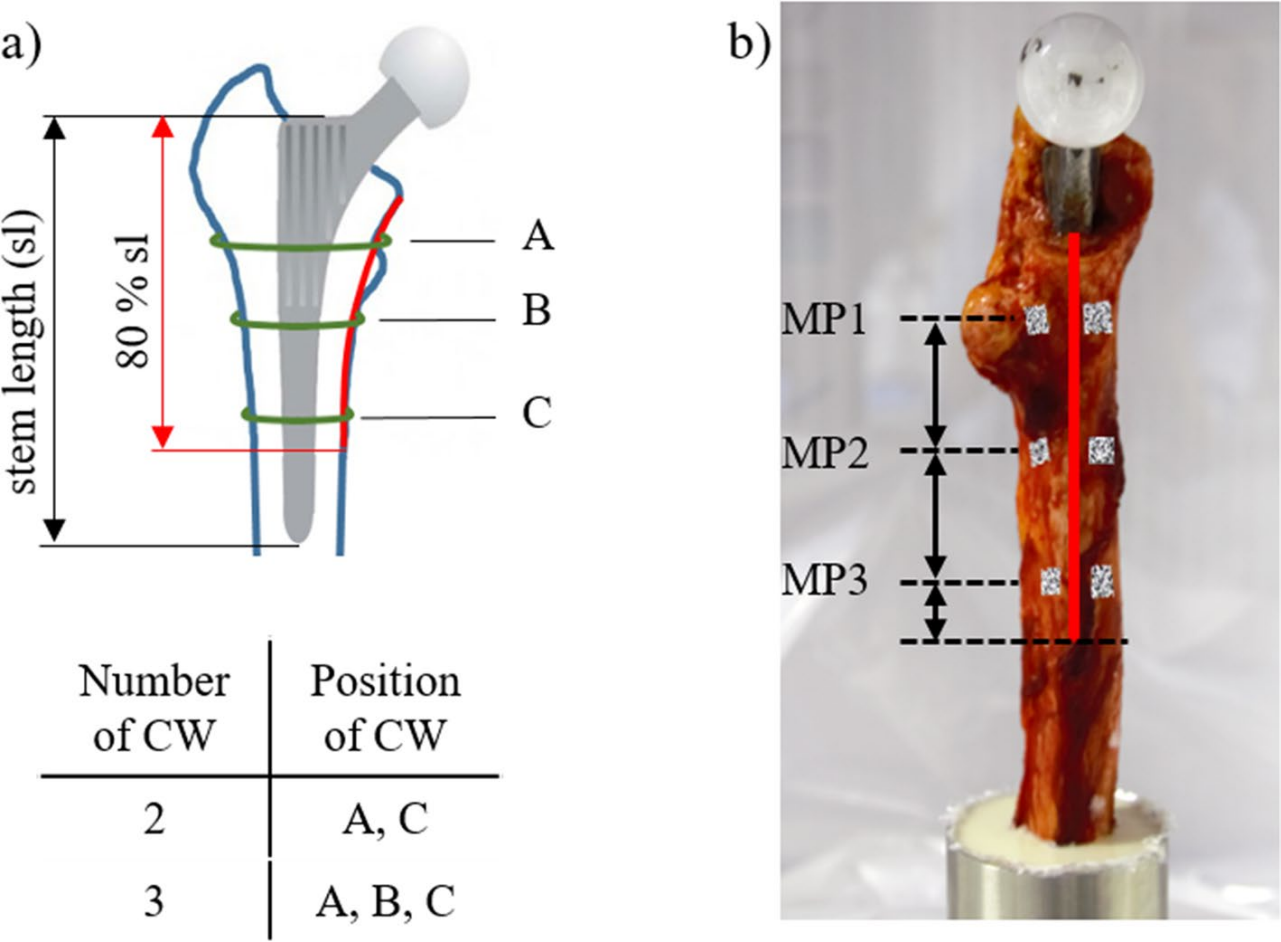
**a** Proximal femur with inserted stem (red: fracture gap, green: cerclage wires). Position A was proximal and position B distal to the lesser trochanter. Position C was 20 mm proximal to the distal end of the fracture gap. **b** Marker positioning of the corresponding measuring point (MP) on the fractured proximal femur: MP 1 was at the level of the lesser trochanter, while MP 3 was 10 mm proximal to the distal end of the fracture gap, and MP 2 was exactly in the middle of MP 1 and MP 3

For all tests, chemically untreated human fresh frozen cadaveric femora were used. The femora were provided by the Institute of Anatomy (Leipzig University). The body donors had given written consent to dedicate their bodies to medical education and research purposes during their lifetime. Being part of the body donor program regulated by the Saxonian Death and Funeral Act of 1994 (3rd section, paragraph 18, item 8), institutional approval for the use of the post-mortem tissues of human body donors was obtained. The Femora were stored at − 80 °*C. prior* to testing, the specimens were analysed using Dual Energy X-ray Absorptiometry (DXA) (Hologic Delphi A QDR-Series, Hologic, Inc., Marlborough, MA, USA) and randomised to different groups (quasi-static, dynamic) including two subgroups (2 CW, 3 CW) each stratified by bone mineral density (BMD). After thawing at room temperature for 24 h, typical femoral Spotorno stems (CBC Evolution, Mathys AG Bettlach, Bettlach, Switzerland) were inserted by a fellowship-trained surgeon. The choice of implant size was made to achieve press-fit. Standardised intraoperative fractures (type II, Modified Mallory Classification) [17] were created propagating medially along the femoral shaft (Fig. 1b). The fracture gap ended distally at 80% of the length of the femoral stem respecting the individual proportions of femur and stem. The fracture was then stabilised using either two or three monofilament CW (1.6 mm, 316 L stainless steel, Synthes, USA; Fig. 1a) which were tensioned and fixed by twisting the ends of the wires. All CW were placed along the entire length of the fracture as previously recommended [18, 19]. Fracture gap was reduced as much as possible [20].

**Fig. 2:**
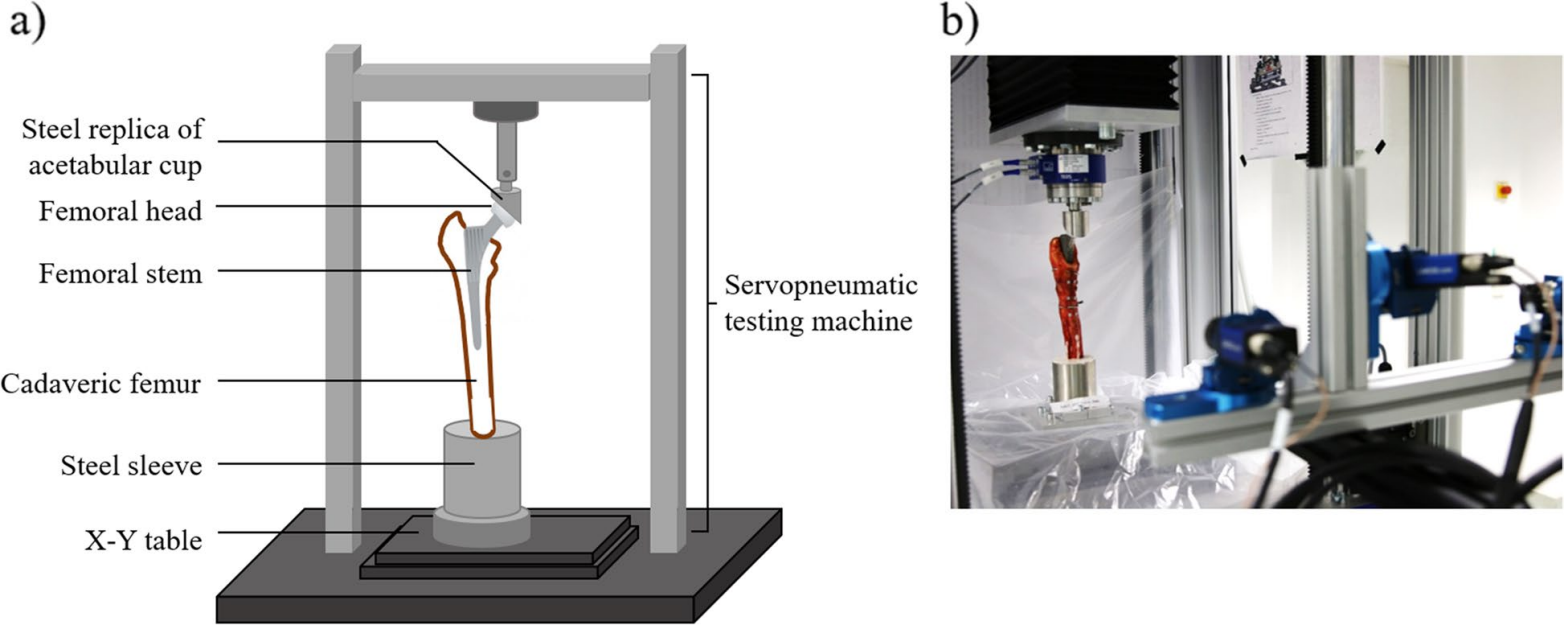
**a** Schematic test setup with construct to be tested. **b** Human cadaveric femur rigidly fixed to the test rig and observed by a 3D camera measuring system

The distal end of the femoral specimen was resected with an oscillating saw 150 mm distal to the end of the stem. The cut of the femoral shaft was made in a way that it was perpendicular to the ideal Mikulicz line (aligning the center of the femoral head to the middle of the knee joint) using a custom-designed gauge. The distal end of the specimen was potted in a steel sleeve (height: 10 mm, diameter: 70 mm) with a quick setting polyurethane system (RenCast FC 52/53, Huntsman Advanced Materials, Basel, Switzerland). For testing, the construct was secured to a fixture on a servopneumatic testing machine (Type 2082/000, DYNA-MESS Prüfsysteme GmbH, Stolberg, Germany) (Fig. 2a). Axial load parallel to the Mikulicz line was applied to an attached ceramic femoral head (diameter: 36 mm; size: L, Mathys AG Bettlach, Bettlach, Switzerland) through a steel replica of the acetabular cup. The femur was aligned using a sliding X-Y table to ensure no transverse forces were applied to the femur at the beginning of the test.

### Quasi-static load testing

Two experimental groups were tested including eleven fractured femora each. In one group, the femora were treated by two CW whereas the femora of the other group were treated by three CW.

The quasi-static load was increased under displacement-control at a rate of 0.2 mm/s until failure (F__failure_). Failure was defined as either audible crack, visible fracture of the femur, failure of at least one CW, or reaching maximum load of the load cell set at 10 kN.

The quasi-static test was designed to prove the effect of constant uniaxial loads on the femur’s stability. In different studies, Bergmann et al. [21–23] provide curves of hip contact forces, measured in vivo in patients during single-leg stance using instrumented total hip implants. Averaged peak loads of 231, 240, and 275.7% body weight (BW) of the corresponding patients were determined. The mean value of the loads provided is approximately 249% BW. Thus, if a specimen resists the defined failure load of 250% BW, sufficient primary stability can be assumed.

### Dynamic cyclic load testing

Two experimental groups consisting of fractured femora were tested. In the experimental group using two CW, two of eleven specimens were excluded because of fractures around the greater trochanter. No specimens were excluded in the other group. Thus, nine specimens were treated by two CW whereas eleven specimens were treated using three CW.

**Fig. 3:**
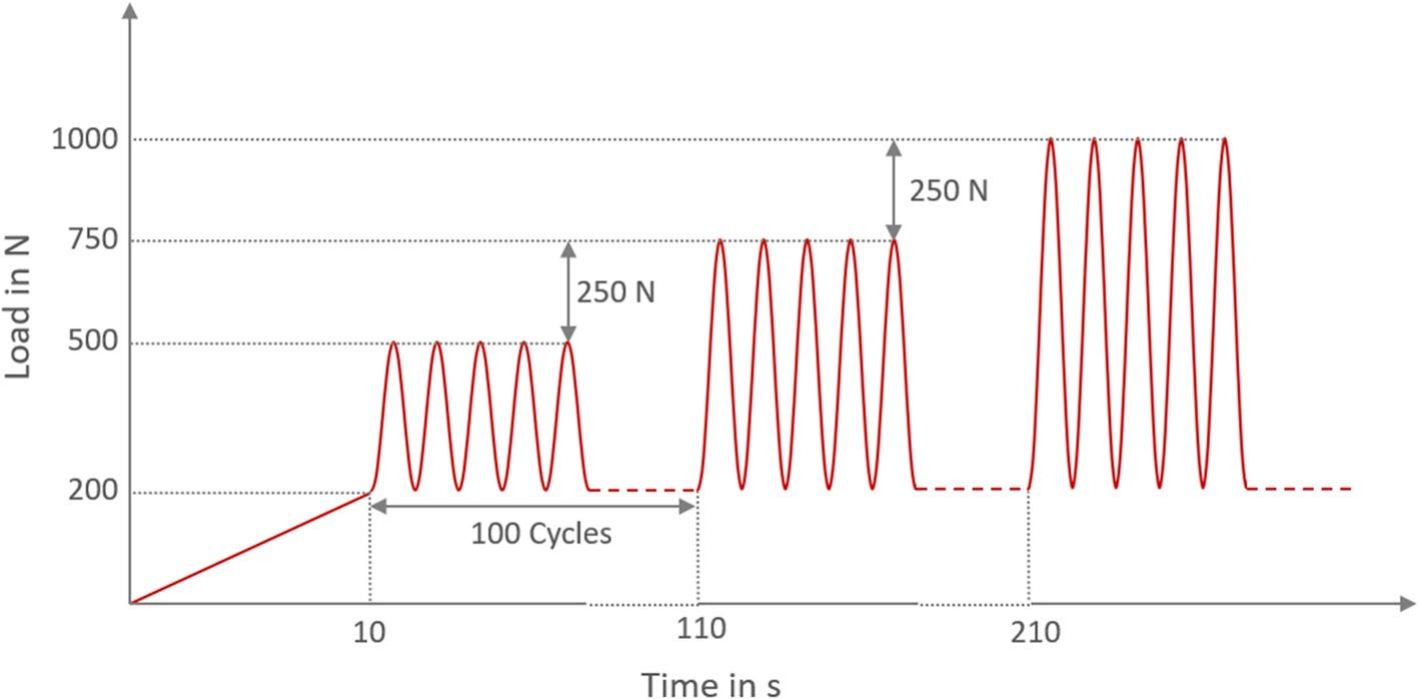
Used cyclic loading protocol

Dynamic cyclic load testing was conducted under the loading protocol shown in Fig. 3. The test consisted of a ramp load of 200 N within the first 10 s and a series of increasing sinusoidal cyclic loads, each with a duration of 100 cycles at a frequency of 1 Hz. While first cyclic loading series ranged between 200 N and 500 N, the maximum load of the following ones was increased by 250 N each while maintaining minimum load of 200 N. The testing procedure was stopped when an audible or visible fracture of the femur occurred, or at least one CW failed. The test was also stopped after 100 cycles with a maximum load of 5250 N, which is known as maximum cyclic physiological load proven by in vivo studies [24].

The dynamic test was designed to quantify the resistance to uniaxial cyclic loading. In contrast to realistic loading conditions, like walking or jogging [24], the direction of load application was constant and no torque was applied throughout the in vitro test. Nevertheless, during walking, mean peak hip contact forces of approximately 250% BW occur similar to those during single-leg stance [22, 23, 25]. Thus, if a specimen resists this load, sufficient primary stability can be assumed.

During the testing procedure, change of fracture gap size was recorded in addition to failure load. For this purpose, each measuring point (MP) at the femur was assigned with a pair of markers (Fig. 1b). The marker displacement was tracked using an image correlation system (Q400, Limess Messtechnik und Software GmbH, Krefeld, Germany) (Fig. 2b). As 200 pictures were taken with a frame rate of 10 fps during the measurement, 20 cycles per cyclic loading series were recorded. After the measurement, spatial marker positions from this image data were calculated. The magnitude of the difference vector of the positions of two adjacent markers was used to define the change of fracture gap size when the construct was loaded or unloaded. This calculation was performed for all three measuring points every 500 N until 5000 N or failure load of the femur was reached. The changes of fracture gap size were averaged over the recorded 20 cycles per cyclic loading series.

### Statistical analysis

Statistical analysis was performed using SPSS 27.0 (IBM Corp., Armonk, New York, USA). Results are reported as mean ± standard deviation. The normality of the data was assessed visually with a histogram and quantitatively with the Shapiro-Wilk test. The statistical significance of difference between two groups was tested using the unpaired Student’s t-test. Statistical significance was set at *p* = 0.05.

## Results

### Quasi-static load testing

**Fig. 4:**
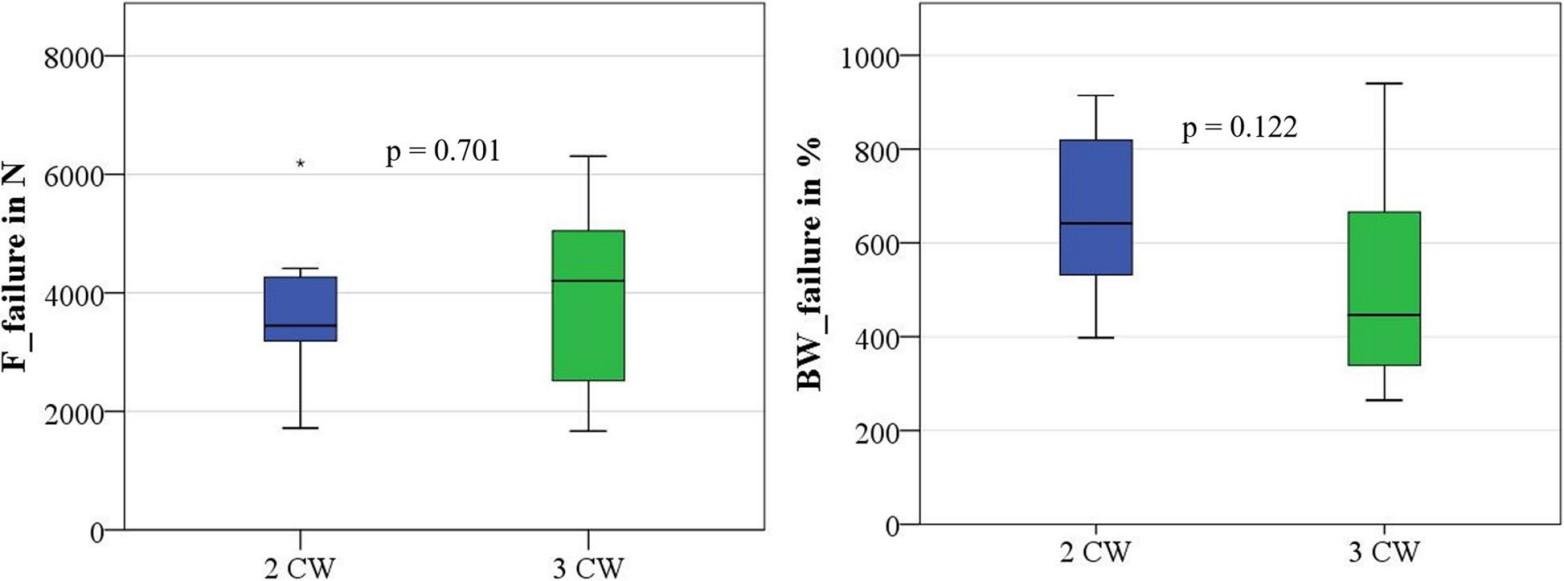
Box plots of failure loads and body weight related failure loads of quasi-static testing groups

Two groups with eleven femoral specimens each were successfully tested and failure loads determined (Supplementary file 1). The results are given in Table 1 and Fig. 4. None of the femora tested resisted the maximum load of the load cell (10 kN). All constructs treated with two or three CW resisted at least 250% BW. The normally distributed failure loads (*p* = 0.701) and BW related failure loads (*p* = 0.122) of the experimental groups were not different.

**Table 1:** Group-specific characteristics (quasi-static load testing)

Group	Number of Males	Body Weight in N	*P*-value	BMD in g/cm^3^	*P*-value	Failure Load in N	Body Weight Failure Load in %
2 CW	6	581.5 ± 158.4	0.027	0.706 ± 0.125	0.675	3727.5 ± 1115.1	658.7 ± 178.2
3 CW	6	809.8 ± 275.7		0.674 ± 0.214		3953.6 ± 1565.0	519.2 ± 223.7

### Dynamic cyclic load testing

**Fig. 5:**
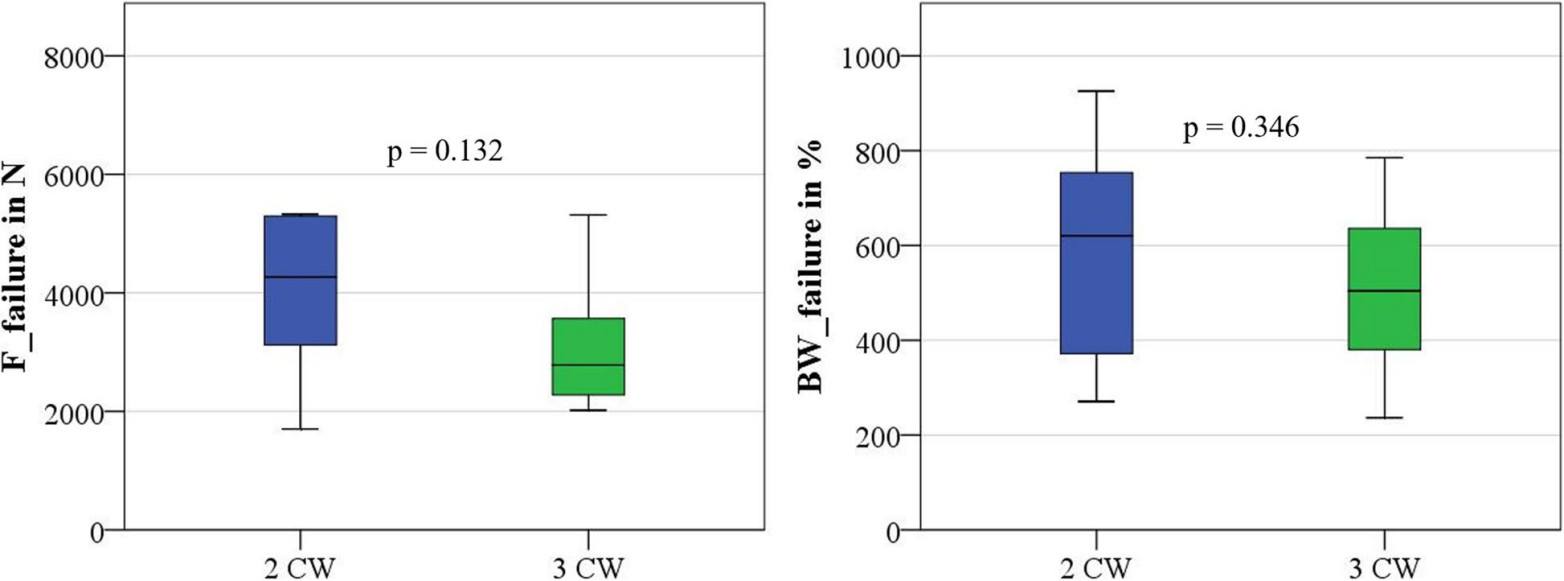
Box plots of failure loads and body weight related failure loads of dynamic cyclic testing groups

In the experimental group using two CW, nine femoral specimens and in the other group eleven femoral specimens were successfully tested and failure loads determined (Supplementary file 2). While two of nine femora tested resisted the predefined maximum load of 5250 N, only one of eleven femora of the other group reached this load. Nevertheless, the normally distributed failure loads (*p* = 0.132) and BW related failure loads (*p* = 0.346) between the groups showed no significant differences. All nine constructs treated with two CW and all but one of the eleven constructs treated with three CW resisted at least 250% BW. The results are given in Table 2 and Fig. 5.

**Table 2:** Group-specific characteristics (dynamic cyclic load testing)

Group	Number of Males	Body Weight in N	*P*-value	BMD in g/cm^3^	*P*-value	Failure Load in N	Body Weight Failure Load in %
2 CW	4	714.0 ± 197.2	0.500	0.670 ± 0.166	0.638	3963.9 ± 1277.8	586.4 ± 227.1
3 CW	5	653.7 ± 192.6		0.705 ± 0.158		3116.1 ± 1125.2	499.7 ± 173.4

**Fig. 6:**
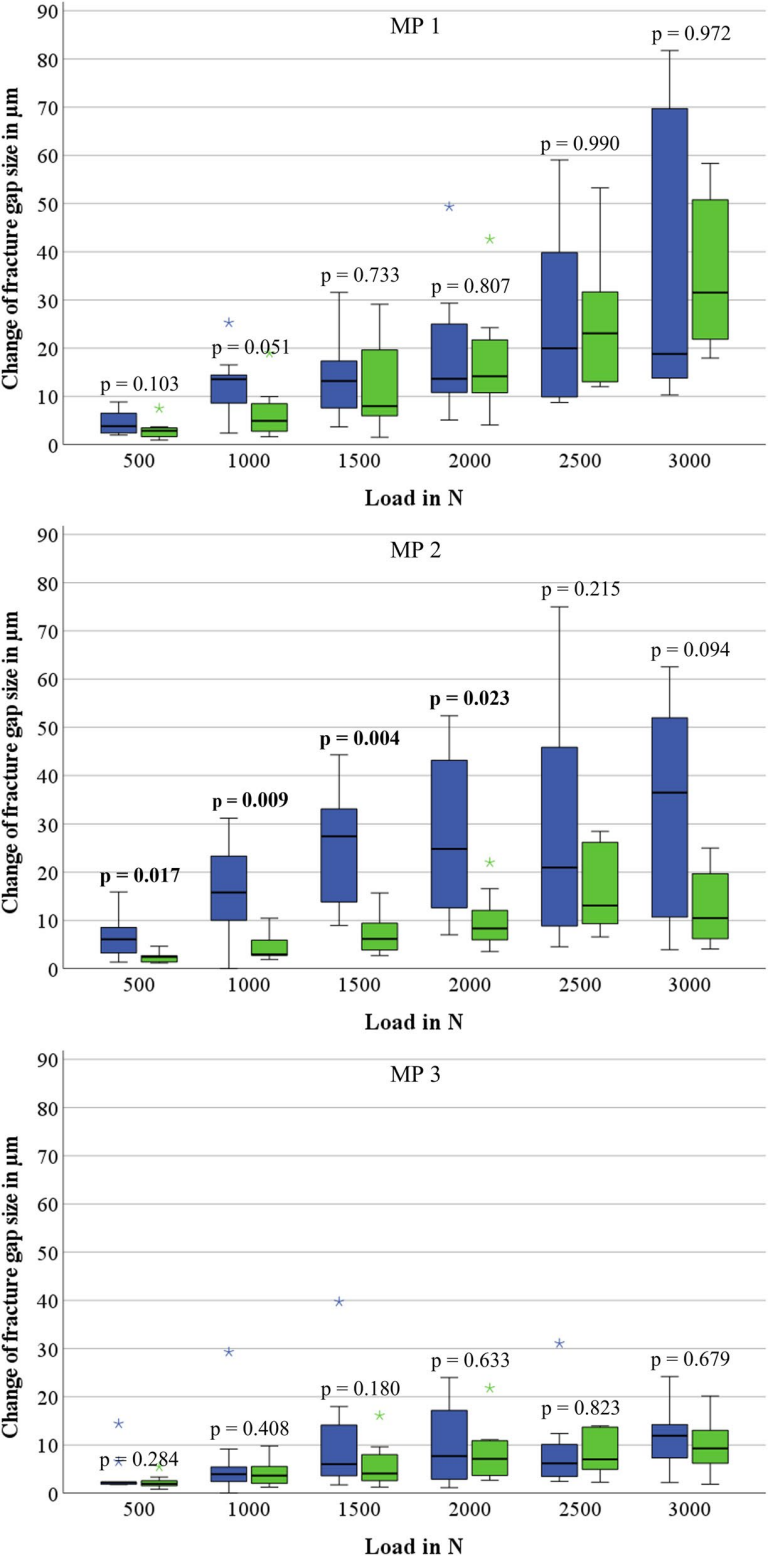
Box plots of load-dependent change of fracture gap size of dynamic cyclic load testing groups regarding MP 1, 2, and 3 (blue: 2 CW, green: 3 CW)

The results of the fracture gap size measurement are shown in Fig. 6. Results are only shown and compared up to a force of 3000 N, as only two specimens of the group using three CW resisted higher loads (Supplementary file 3). At MP 1, the fracture gap widened with increasing force in both groups but showed no significant differences at all times. The same applies to MP 3, as there were no significant differences between the groups. At MP 2, greater changes of gap size were observed in the group using two CW. While the changes of fracture gap size were significantly higher from 500 N to 2000 N, the groups did not differ significantly when a higher load was applied.

## Discussion

With the increasing number of THA and due to the trend towards uncemented stem fixation, the incidence of IPFF increases [5, 6]. Fracture treatment aims to achieve fixation with high primary stability allowing early full weight-bearing. This study aimed to analyse the primary stability of IPFF fixation using different numbers of CW. Therefore, quasi-static and dynamic cyclic loading tests were performed on human cadaveric femora determining failure loads. Additionally, in case of dynamic loading tests, the change of fracture gap size was optically captured to determine crack opening while loading.

Some authors suggest cerclage wiring should only act as adjunct to nail or plate fixation [26–28]. In some cases, however, the authors refer to fracture types other than the one considered in this study. Perren et al. stated insufficient mechanical stability due to the treatment of CW, but they only consider type C fractures according to the Vancouver classification [28]. Other authors such as Agarwala et al. also reported inadequate stability when using CW but they do not specify the fracture type or evidence supporting their thesis. In contrast, Park et al. proved very good results of calcar crack treatment using CW based on fixation’s sufficiently high primary stability [29]. Fishkin et al. also concluded in their biomechanical study that cerclage wiring can lead to sufficiently high primary stability needed for patient’s early mobilisation [19]. Their results demonstrate the superiority of two or three CW compared to the use of only one CW based on measurements of the fracture gap size at discrete loads. Therefore, they recommend the usage of at least two CW treating femoral cracks. In accordance to Fishkin et al., the results of the present study show that the fixation of standardised IPFF (type II, Modified Mallory Classification) using two or three CW provides sufficient primary stability against uniaxial loads.

To the best of our knowledge, the only comparable biomechanical study was carried out by Frisch et al. [30]. They also examined different cerclage fixation techniques. However, in contrast to the present study, they used artificial bones, applied quasi-static axial loads and quasi-static torques. Their used monofilament cerclage wires showed a similar mean failure load (4010 N) in comparison to our study (3727.5 N). However, the comparability of artificial bones and human bones is severely limited because the artificial bones are more stable than the commonly used human bones of geriatric patients. They rather simulate the mechanical behaviour of young, healthy bones [31].

As no significant differences could be found neither in the quasi-static nor in the cyclic test, and only one sample withstood less than 250% BW in all tests performed, it can be assumed that both cerclage wiring techniques provide sufficient primary stability according to axial peak hip contact forces simulating walking or single-leg stance. However, proof of application necessity of three CW was not possible. With regard to the increasing risk of complications related to the use of additional CW, like injury of blood vessels [16, 32] or bone surface resorption [15], as least CW as required should be used. Another advantage of using lesser CW is the shortening of procedure time [33]. Therefore, in the authors’ opinion, fracture treatment using two CW is to prefer.

Nonetheless, this study has several limitations. As a major limitation, it should be noted that only axial loads were analysed. In accordance with other in vitro studies using uniaxial testing machines [19, 30], we applied loads of only one direction. Thus, we only determined specimens’ resistance to one force direction. In dynamic activities like walking the direction of the hip contact force changes during the gait cycle [23]. In addition, activities like this are characterised by occurring torque loads. However, we did not combine the axial loads with these occurring torque loads. Therefore, no statement can be made about the torsion stability of the fixation technique used. Further, study results are only valid for the stems tested. Another limitation to this study was the low sample size. Although the sample size was comparable to previous biomechanical studies of proximal femoral fractures [19, 34], it was nevertheless small. That we were unable to identify a significant advantage of one fixation technique may be a consequence of our limited sample size.

It is noticeable that the change of fracture gap size generally is at a very low level (< 100 μm) during dynamic cyclic loading. There were no differences between the two fixation techniques at the proximal MP 1 and the distal MP 3. At MP 2, fractures treated by only two CW showed significantly higher movements recognisable by larger changes in fracture gap size. As described in the literature, a positive effect on callus formation can be assumed due to higher interfragmentary movements within this area [20, 35, 36]. Considering that, size of movement should be in a range of 0.2 mm to 1.0 mm [37]. The determined changes in fracture gap size were below this range, thus no positive effect is assumed due to the increased movement.

In contrast, the small movements determined have a favourable impact on prosthesis’ osseointegration. Due to the geometric conditions, it can be assumed that the largest movement between prosthesis and bone occurs at the fracture gap. Therefore, the micromotions of the bone-implant interface are below 100 μm. In literature it is mentioned, that below the threshold of 150 μm bone formation occurs on the bone-implant interface [38]. Consequently, osseointegration should not be threaten by the movements at the fracture gap.

## Conclusions

IPPF are a relevant complication following THA, which mainly occurs in elderly patients. Early mobilisation is desirable to prevent risks such as thrombosis, embolism or pneumonia. Therefore, the aim is to achieve a sufficiently high primary stability to allow early weight-bearing and daily rehabilitation exercises without extensive treatments like stem changes or plate osteosynthesis. This in vitro biomechanical study showed, that both two and three CW provide sufficient primary stability for fixation of intraoperative proximal femoral fractures according to the predefined minimum failure load (250% BW) to resist. In the authors’ opinion, there are several advantages of the fracture fixation using only two CW like procedure time reduction and risk minimization of vascular injury.

## Supplementary Information

**Additional file 1.**

**Additional file 2.**

**Additional file 3.**

## Data Availability

All data generated or analysed during this study are included in this published article and its supplementary information files.

## Abbreviations

BMD: Bone mineral density
BW: Body weight
CW: Cerclage wires
IPFF: Intraoperative proximal femoral fracture
MP: Measuring point
SL: Stem length
THA: Total hip arthroplasty
